# Comparison of the effects of Tai Chi and general aerobic exercise on weight, blood pressure and glycemic control among older persons with depressive symptoms: a randomized trial

**DOI:** 10.1186/s12877-022-03084-6

**Published:** 2022-05-07

**Authors:** Yan Wang, Biru Luo, Xiaoqin Wu, Xiaoxia Li, Shujuan Liao

**Affiliations:** 1 Department of Nursing, West China Second University Hospital, Sichuan University, Chengdu, 610041 China; 2 Key Laboratory of Birth Defects and Related Diseases of Women and Children (Sichuan University), Ministry of Education, Sichuan University, Chengdu, 610041 China; 3 The People’s Hospital of Jiawang District of Xuzhou, Xuzhou, 221011 China; 4 Ya’ an Polytechnic College, Ya’ an, 625100 China

**Keywords:** Tai Chi, Aerobic exercise, Older person, Blood pressure, Blood glucose, Randomized

## Abstract

**Background:**

Blood pressure and glycemic control are associated with the management of depressive symptoms in patients with depression. Previous studies have demonstrated that both Tai Chi and aerobic exercise have positive effects on blood pressure and glycemic control. Few studies have compared the physiological effects of Tai Chi versus aerobic exercise in older adults with depressive symptoms. The objective of this study was to compare the effects of Tai Chi and aerobic exercise on weight, body mass index, blood pressure and glycosylated hemoglobin (HbA1c) level in older persons with mild to moderate-severe depressive symptoms.

**Methods:**

A randomized controlled trial was performed. The older persons (age ≥ 60 years old) with depressive symptoms were recruited. Then, participants were randomly allocated to the Tai Chi group and the aerobic exercise group received a 12-week 24-movement Yang’s Tai Chi intervention and aerobic exercise, respectively. Data collection occurred at baseline and after completion of the interventions (week 12).

**Results:**

A total of 238 participants with mild to moderate-to-severe depressive symptoms were included in the final analysis, including 120 in the Tai Chi group and 118 in the aerobic exercise group. The difference in weight and body mass index in the Tai Chi group was 2.0 kg (*Z* = -4.930, *P* < 0.001) and 0.77 kg/m^2^ (*Z* = -5.046, *P* < 0.001) higher than that in the aerobic exercise group, respectively. After the 12-week intervention, the systolic pressure and diastolic pressure in the Tai Chi group were 5.50 mmHg (*Z* = -2.282, *P* = 0.022) and 8.0 mmHg (*Z* = -3.360, *P* = 0.001) lower than that in the aerobic exercise group, respectively. The difference in HbA1c level in the Tai Chi group was 0.50% higher than that in the aerobic exercise group (*Z* = -4.446, *P* < 0.001).

**Conclusion:**

This study showed that Tai Chi exercise was more effective in improving blood pressure and HbA1c level than general aerobic exercise. It suggested that Tai Chi might be an effective approach for the management of blood pressure and long-term glucose control in older persons with depressive symptoms.

**Trial registration:**

Trial registration: ChiCTR, ChiCTR2100042534. Registration date: 23/01/2021, http://www.chictr.org.cn/showproj.aspx?proj=120602.

## Background

Tai Chi is a form of mind–body exercise usually described as “meditation in motion”. This exercise developed as an ancient Chinese martial art [1] and is today becoming popular in many Asian countries such as South Korea, Japan and Western countries such as Canada, Spain, Australia, and the United States. In the United States, more than 2 million people practice Tai Chi [2]. Tai Chi is generally considered as a mind–body exercise which combines physical activity, breath inspiration and expiration, and mind regulation altogether [1]. Differ from general aerobic exercise, Tai Chi aims to achieve physical and mental unity through three key points of regular motion of the whole body, breathing regulation and mental concentration during the practice [3].

Tai Chi is widely practiced in the older population because of the slow and gentle movements. The safety of Tai Chi has also been demonstrated among older dwellers [3], they could even learn and practice at home without supervision. Moreover, Tai Chi is beneficial in improving older person’s physical and psychological well-being. Evidence from clinical data have identified Tai Chi plays an important role in improving chronic diseases such as hypertension [4], chronic obstructive pulmonary disease and chronic heart failure [5], preventing falls [6], controlling blood glucose [7], and alleviating psychological symptoms such as depression and anxiety [8–10] in the old peoples.

Exercise has been found to be effective in reducing depressive symptoms [11]. Evidence suggests that moderate-intensity aerobic exercise with the frequency of three times a week for at least nine weeks has a positive therapeutic effect on depression [12]. As a specific aerobic exercise, randomized trials have shown that Tai Chi has a significant effect on reducing depressive symptoms compared to the blank control or health education groups [13]. Our previous studies implemented a 12-week Tai Chi intervention among community-dwelling older persons with mild to moderate depressive symptoms, also revealed that Tai Chi could significantly improve their depressive symptoms and quality of life compared with blank control [8, 14]. Other systematic reviews and randomized controlled trials also demonstrated that Tai Chi had a positive effect on improving depressive symptoms in older persons [10, 13, 15].

Although promising findings have been reported regarding to the psychologic effect of Tai Chi, few studies have compared its physiological benefits such as blood pressure (BP) and indicators of glycemic control versus conventional exercise, such as aerobic exercise in older adults with depressive symptoms. BP and glycemic control are associated with the management of depressive symptoms. Patients with clinically significant symptoms of depression were more likely to suffer from hypertension [16] and experience poor glycemic control (i.e., higher glycosylated hemoglobin) [17]. Previous studies have demonstrated that Tai Chi had positive effects on improving blood pressure and glycemic control [18, 19]. Therefore, the objective of this study was to compared the effects of Tai Chi and aerobic exercise on physiological conditions including weight, body mass index, BP and glycosylated hemoglobin (HbA1c) level in older persons with mild to moderate-severe depressive symptoms. We hypothesized that Tai Chi was more effective in the management of blood pressure and glycemic among older persons with mild to moderate-severe depressive symptoms compared with traditional aerobic exercise.

## Methods

### Aim and design

We aimed to compare the effects of Tai Chi and aerobic exercise on weight, body mass index, blood pressure and glycosylated hemoglobin (HbA1c) level in older persons with mild to moderate-severe depressive symptoms. A randomized controlled trial was conducted.

### Participants

Cluster sampling method was used in this trial to avoid contamination. Six communities were randomly selected from 24 communities in Ya’ an City, then the selected communities were assigned to either the Tai Chi group or the aerobic exercise group according to random numbers generated by Microsoft Excel software version 2016. Older persons who met the inclusion criteria were selected as the study subjects in each community. Either a 12-week Yang’s Tai Chi or aerobic exercise intervention was offered to older persons with depressive symptoms from these six communities depending on their allocation. Older persons were eligible if they: had mild to moderate-severe depressive symptoms; aged 60 years old and above; did not have any other mental illness; had lived in the community for at least 1 year. Older persons were excluded if they: were not suitable for Tai Chi and aerobic exercise, such as severe vision or hearing impairment; had a history of cardiovascular disease or stroke in the past 6 months; were accepting antidepressants; exercised regularly at a frequency of equal to and more than 3 times per week.

Depressive symptoms were assessed by the Patient Health Questionnaire 9-Item (PHQ-9), which was initially developed by Kroenke [20]. It was a self-rating scale for the depression-related symptoms experienced in the past 2 weeks, scoring each item on a four-point Likert scale of 0–3, with 0 indicating “not at all” and 3 indicating “almost every day”. PHQ-9 is widely used in both clinic and community settings. It was introduced to China in 2007 [21]. The reliability and validity of PHQ-9 were demonstrated in the elderly populations in mainland China [22]. The cut points of 5, 10, 15, and 20 were interpreted as representing mild, moderate, moderate-severe, and severe levels of depression on the PHQ-9 [20].

### Intervention

Tai Chi and aerobic exercise interventions were carried out simultaneously to eliminate potential confounding from seasonal influences on symptom severity. Tai Chi intervention was offered 3 times per week for 12 consecutive weeks, and each session lasted 60 min, which was demonstrated to be the most popular approach in previous studies [23]. Likewise, aerobic exercise intervention was offered 3 times per week for 12 consecutive weeks, each session lasted 60 min. Attendance forms and sign-in sheets were used to monitor attendance of each participant at intervention lessons. Study subjects were allowed to take one day off a month, and participants who missed two or more times a month were excluded. Participants in both Tai Chi and aerobic exercise groups received a closely supervised, group-format intervention sessions in their community's outdoor plaza. Two to four research assistants who were responsible for supervising and observing participants to avoid accidents, were scheduled for each intervention session.

#### Tai Chi

24-Style Yang’s Tai Chi was adopted in the study. The 24-Style Tai Chi is simplified from classical 108-movement Yang style Tai Chi, which was more suitable for older persons to learn and practice within 12 weeks and was also the most common style adopted in published literature. The key points of Tai Chi were regular motion of the whole body, breathing regulation and mental concentration during the practice. Tai Chi intervention session was leaded by 3 instructors who had extensive experience of 20 years and over. Moreover, all 3 instructors had been trained for study subject protection at the beginning of the intervention classes. Safety precautions were informed to all participants. The theory and procedures of Tai Chi were explained by the instructor at the first session to ensure that participants grasp the essentials of Tai Chi exercise. Learning and reviewing occurred in the consequent sessions. The total duration of Tai Chi exercise was about 60 min.

#### Aerobic exercise

Participants allocated to aerobic exercise group received a 20-movement low impact aerobics. The 20-movement exercise consisted of several components: shoulder movement, arm movement, chest expand, waist movement, and leg movement. These 20 movements were low-intensity and were mainly dynamic stretching. Each movement took 1 min. The 20-movement aerobic exercise cycled for 3 times during each session. The total duration of Tai Chi exercise was about 60 min. Three nursing students conducted aerobic exercise intervention as instructors. Before the intervention began, the three nursing students were trained about the 20 movements and the subject protection procedure. Moreover, safety precautions were told to all participants. The aerobic exercise sessions were strictly monitored by research assistants to ensure participants’ comfort and safety and to minimize adverse events.

### Outcomes measures and follow up

Participants’ sociodemographic data was collected at baseline using a self-designed form, including age, marital status, living status, education years, living aera, and number of diseases. The outcomes of the study were weight, BMI, BP and HbA1c level, which were assessed at baseline and after completion of the interventions (week 12). Height and weight were self-reported by participants. Body mass index (BMI) was calculated as weight (kg) divided by height (m) squared. BP was assessed by mercurial sphygmomanometer (YUWELL, 20,152,200,947). BP of each participant was measured three times after a 30-min rest, and the average of the three measurements was taken as the final value. Hypertension was defined as a systolic blood pressure (SBP) of 140 mmHg or greater or a diastolic blood pressure (DBP) of 90 mmHg or greater. Peripheral blood was collected by EDTA tube before intervention and HbA1c was assessed after 12-week intervention.

### Sample size

Sample size was calculated based on the results of one previous meta-analysis [13]. The hypothesized was that Tai Chi was more effective than aerobic exercise. A two-sided hypothesis test at 0.05 significance level with an allocation ration of 1:1 was used, for a 90% statistical power to detect a significant effect of Tai Chi over aerobic exercise, a 20% attrition rate was estimated. A sample size of 240 (120 in the Tai Chi group, 120 in the aerobic exercise group) was finally determined.

### Randomization

Six communities were selected from 24 communities and assigned to the Tai Chi group and aerobic exercise by random number table method. Microsoft Excel (version 2016) was used to generate table of random numbers with “1” and “2”. The number “1” was set to appear six times and the number “2” was set to appear 18 times. Six communities corresponding to the number “1” were included, while 18 communities corresponding to the number “2” were excluded. Then, 3 communities corresponding to the number “1” were assigned to the Tai Chi group and 3 communities corresponding to the number “2” were assigned to the aerobic exercise group by using the same procedure.

Qualified older persons in the six communities were considered as candidate participants of our study. After screening for eligibility, candidates were recruited by the same random number table method mentioned above, according to the proportion determined by the sample size and qualified population base of each community. Microsoft Excel (version 2016) was used to generate table of random numbers with “1” and “2”. Participants who corresponded to number “1” were included, while participants corresponding to number “2” were excluded.

### Blinding

The data collectors, physical indicators assessors, and laboratory tester who conducted the baseline and follow-up assessments were blinded to treatment allocation.

### Data analysis

The qualitative data was described by frequency and proportion, and the data were statistically inferred by chi-square test. The quantitative data was statistically described by mean and standard deviation (*SD*), and the data were statistically inferred by independent t-test. A two side *P* value < 0.05 was considered to indicate significant differences. All analyses were performed in Stata version 16.0.

## Results

### Participant recruitment and retention

**Fig. 1:**
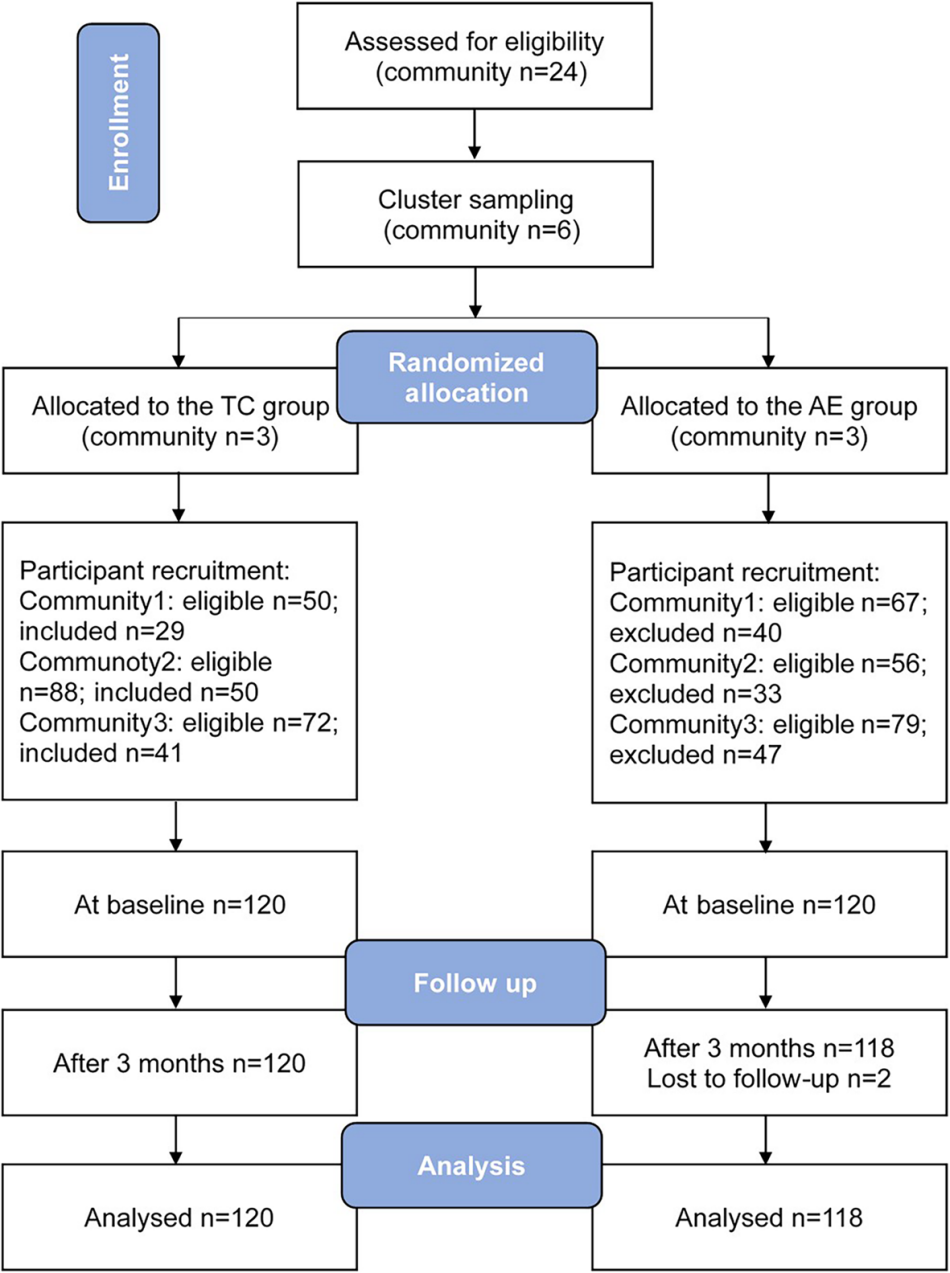
CONSORT flow diagram of participant recruitment and retention. TC = Tai Chi; AE = aerobic exercise

A total of two hundred and forty older persons with depressive symptom were enrolled, including 120 in the Tai Chi group and 120 in the aerobic exercise group. Two participants in the aerobic exercise group were lost to follow up after 12-week intervention. A total of 120 participants in the Tai Chi group and 118 participants in the aerobic exercise group were included in the final analysis. The number of retaining participants in each community were showed in CONSORT flow diagram (Fig. 1).

### Baseline characteristics of participants

There were no differences in sociodemographic characteristics of older persons in the Tai Chi group and in the aerobic exercise group. The ages of participants ranged from 60 to 85 years old, with 125 female participants (52.25%). The baseline characteristics are summarized in Table1.

**Table 1:** Sociodemographic characteristics of participants

Characteristics	Overall (*n*=238)	Tai Chi (*n*=120)	Aerobic exercise (*n*=118)	*t/χ*^2^*/Z*	*P* value
Age (year)	68.0 (10.0)	69.0 (9.0)	67.0 (12.0)	0.940	0.349
Height (cm)	158.0 (12.0)	158.0 (10.0)	158.0 (14.0)	-0.344	0.731
Weight (kg)	60.0 (13.0)	60.0 (13.0)	60.0 (14.3)	-0.705	0.481
BMI (kg/m^2^, *Mean±SD*) ^a^	23.26±3.35	23.54±3.74	22.97±2.89	1.297	0.196
Gender n (%)				1.092	0.296
Male	113 (47.68)	61 (50.83)	52 (44.07)		
Female	125 (52.52)	59 (49.17)	66 (55.93)		
Marital status n (%)				3.21	0.073
Married	195 (81.93)	93 (77.50)	102 (86.44)		
Single (Unmarried/Divorced/ Separated/Widowed)	43 (18.07)	27 (22.50)	16 (13.56)		
Living status n (%)				5.176	0.395
Living with partner	100 (42.02)	43 (35.83)	57 (48.31)		
Living with partner and children	65 (27.31)	33 (27.50)	32 (27.12)		
Living with children	43 (18.07)	26 (21.67)	17 (14.41)		
Living with caregiver	2 (0.84)	1 (0.83)	1 (0.85)		
Live alone	25 (10.50)	15 (12.50)	10 (8.47)		
Other	3 (1.26)	2 (1.67)	1 (0.85)		
Education years (year)				6.829	0.078
0-6	55 (23.11)	33 (27.50)	22 (18.64)		
6-9	95 (39.92)	51 (42.50)	44 (37.29)		
9-12	71 (29.83)	27 (22.50)	44 (37.29)		
>12	17 (7.14)	9 (7.50)	8 (6.78)		
Per capita monthly income (yuan)				0.589	0.443
<=1499	143 (60.08)	75 (62.50)	68 (57.63)		
>1499	95 (39.92)	45 (37.50)	50 (42.37)		
Number of diseases n (%)				2.002	0.572
0	46 (19.33)	24 (20.00)	22 (18.64)		
1	77 (32.35)	42 (35.00)	35 (29.66)		
2	50 (21.01)	21 (17.50)	29 (24.58)		
>=3	65 (27.31)	33 (27.50)	32 (27.12)		
Per capita living area (m^2^)	25.0 (20.0)	25.0 (20)	20.5 (18.0)	-1.279	0.201

*Note*: *SD* Standard deviation, *BMI* Body mass index

^a^ BMI was described as *Mean±SD*, other variables were described as median and inter-quartile

Independent t-test, chi-square test and Mann–Whitney U test were used

### Comparison of weight and weight change

There was no difference in weight and BMI between the Tai Chi group and the aerobic exercise group at baseline and after the 12-week intervention (Table 2). However, the weight change and BMI change in the Tai Chi group was 2.0 kg (*Z* = -4.930, *P* < 0.001) and 0.77 kg/m^2^ (*Z* = -5.046, *P* < 0.001) higher than that in the aerobic exercise group, respectively.

**Table 2:** The effects of Tai Chi on weight and BMI change of participants

Variables	All (*n* = 238)	Tai Chi group (*n* = 120)	Aerobic exercise group (*n* = 118)	*t/Z*	*P* value
Weight (kg)					
At baseline	60.0 (13.0)	60.0 (13.0)	60.0 (14.3)	-0.705	0.481
Week 12	55.0 (10.0)	55.0 (10.0)	55.0 (10.0)	-0.366	0.714
Weight difference	3.0 (3.0)	4.0 (3.0)	2.0 (2.0)	-4.930	< 0.001
BMI (kg/m^2^*)*					
At baseline (*Mean* ± *SD*) ^a^	23.26 ± 3.35	23.54 ± 0.34	22.97 ± 0.27	1.297	0.196
Week 12	21.85 (3.71)	21.48 (4.03)	22.16 (3.31)	-0.616	0.538
BMI difference	1.06 (1.10)	1.60 (1.15)	0.83 (0.80)	-5.046	< 0.001

Note: *SD* Standard deviation, *BMI* Body mass index

^a^ BMI (at baseline) was described as *Mean* ± *SD*, other variables were described as median and inter-quartile

Independent t-test and Mann–Whitney U test were used

### Comparison of blood pressure and HbA1c level

There was no difference in SBP, DBP, and HbA1c level between the two groups at baseline. After the 12-week intervention, the SBP and DBP in the Tai Chi group was 5.50 mmHg (*Z* = -2.282, *P* = 0.022) and 8.0 mmHg (*Z* = -3.360, *P* = 0.001) lower than that in the aerobic exercise group, respectively. The HbA1c level in the Tai Chi group was lower than that in the aerobic exercise group. The difference of HbA1c level in the Tai Chi group was 0.50% higher than that in the aerobic exercise group (*Z* = -4.446, *P* < 0.001). Detailed information is shown in Table 3.

**Table 3:** The effects of Tai Chi on blood pressure and HbA1c of participants

Variables	All (*n* = 238)	Tai Chi group (*n* = 120)	Aerobic exercise group (*n* = 118)	*t*/χ^2^/Z	*P* value
Hypertension n (%)					
At baseline	111 (46.64)	54 (45.00)	57 (48.31)	0.261	0.609
Week 12	111 (46.64)	54 (45.00)	57 (48.31)	0.261	0.609
Systolic pressure (mmHg)					
At baseline (*Mean* ± *SD*) ^a^	132.90 ± 16.53	131.53 ± 15.89	134.30 ± 17.12	-1.295	0.197
Week 12	121.50 (30.0)	120.0 (30.0)	125.50 (22.0)	-2.282	0.022
Difference	8.0 (7.0)	9.50 (6.0)	6.0 (10.0)	-3.527	< 0.001
Diastolic pressure (mmHg)					
At baseline	81.0 (15.0)	80.0 (15.0)	82.0 (14.0)	-1.404	0.160
Week 12	75.0 (18.0)	71.0 (15.0)	79.0 (8.0)	-3.360	0.001
Difference	4.50 (9.0)	6.0 (8.0)	4.0 (6.0)	-3.887	< 0.001
HbA1c (%)					
At baseline	7.90 (2.90)	7.80 (4.0)	7.90 (2.20)	-0.392	0.695
Week 12	7.55 (2.80)	7.10 (3.0)	7.70 (2.30)	-2.084	0.037
Difference	0.40 (1.0)	0.70 (1.0)	0.20 (0.40)	-4.446	< 0.001

Note: *SD* Standard deviation, *HbA1c* Glycosylated Hemoglobin

^a^ Systolic pressure (at baseline) was described as *Mean* ± *SD*, other variables were described as median and inter-quartile

Independent t-test, chi-square test and Mann–Whitney U test were used

### Adverse events

No adverse events were observed in the experimental period.

## Discussion

The findings of this study showed that the 12-week community-based Tai Chi intervention was more effective in weight reduction, improving BP and HbA1c level in older persons with depressive symptoms compared to low impact aerobics.

Our study found there was no significant difference in weight and BMI between the two groups after the 12-week intervention, which was supported by a few studies [24, 25]. A recent three-group trials showed a 12-week Tai Chi program had no significant effects on weight and BMI from baseline to week 12 and 38 compared with conventional exercise [25]. However, our result was also inconsistent with some studies [26–28]. Sun and Buys [26] demonstrated that Tai Chi was effective in BMI reduction in Chinese older adults with a 12-months intervention. Ma [28] found a 6-months group-based Tai Chi training combining home practice was beneficial in improving BMI among community-dwelling older adults. A 6-years follow-up study showed that long-term Tai Chi exercise was significantly effective in reducing BMI compared to those in the control group [27]. The effectiveness of Tai Chi in weight and BMI may be explained by the important role of Tai Chi in metabolism balance of human body and energy consumption of gas and on body sculpting and fitness [5, 29]. The potential reasons for the discrepancies in the effectiveness of Tai Chi in weight loss and BMI reduction reported in our study compared with other studies were the differences in the participants and the duration of intervention. Our study involved older adults with depressive symptoms, while participants in other studies were older adults with obesity or hypertension. Exercise is more effective in obese people in reducing weight and BMI. Additionally, our study only intervened for 12 weeks, which was less than other studies. However, our study found that changes in weight and BMI in the Tai Chi group were significantly greater than those in the aerobic exercise group. It suggested Tai Chi exercise may be more effective in reducing weight and BMI than general aerobic exercise. Prolonging the duration of intervention may be effective in improving body weight and BMI.

In this study, SBP and DBP were significantly reduced after Tai Chi exercise by 5.50 mmHg and 8.0 mmHg after the 12-week intervention, respectively, as compared to the low impact aerobics in the old people with depressive symptoms. Additionally, the difference in SBP and DBP before and after Tai Chi intervention were 9.50 mmHg and 6.0 mmHg, which were significantly higher than that before and after low impact aerobics intervention. This result was consistent with previous study [28]. Ma [28] found Tai Chi significantly lowered BP than the usual care (UC) group in the older people. Tai Chi also has been shown to reduce BP in other populations. A systematic review summarized Tai Chi exercise can lower SBP by 7 mmHg to 32 mmHg in subjects with hypertension, cardiovascular populations and non-cardiovascular populations and healthy peoples [18]. However, our result was inconsistent with some studies [25]. One systematic review reported that two RCTs of Tai Chi failed to show a reduction of resting BP compared with aerobic exercise, low impact exercise and no exercise control [30]. A recent three-arm trial conducted in middle-aged and older adults found there was no significant difference in SBP and DBP between Tai Chi exercise and conventional exercise [25]. The reduction in BP values varies across studies. This may be related to the clinical characteristic of the participants and the intensity, dose, and style of the Tai Chi exercise. This study provided more clinical data on the antihypertensive effects of Tai Chi. Regular exercise may be recommended for people with normal blood pressure, prehypertension, and hypertension to prevent the development of hypertension [31].

Significant improvement in HbA1c level was found between preintervention and postintervention time in the Tai Chi group compared to the aerobic exercise group among the old people with depressive symptoms. Our finding was consistent with Zhou’s study, which synthesized data from 23 studies and found significant changes in Tai Chi-related effects were observed in lowering HbA1c (mean difference –0.88%; 95% CI –1.45% to –0.31%; *P* = 0.002) [19]. This result was controversial with another meta-analysis, which considered subgroup analysis and showed that Yang-style Tai Chi did not significantly reduce HbA1c after a duration ≤ 3 months or > 3 months. However, other styles of Tai Chi significantly reduced HbA1c with a interventional duration > 3 months, while no reduction was found after a duration ≤ 3 months [32]. The difference may be related to different training durations and styles of Tai Chi. It suggested that the dosage and type of Tai Chi were related to its effect size of glycemic control. HbA1c effectively reflect average blood glucose level in the past 8–12 weeks and is an indicator of long-term glucose control. Our study showed that the 12-week Tai Chi program reduced participants' HbA1c by 0.60%, which were at a moderate level compared with previous studies [33–35]. It suggested our 12-week Tai Chi program may be effective on long-term glucose control of older persons.

Our study had some limitations. First, the duration of intervention was only 12 weeks in this study, lower than 6 months,12 months or even 2 years in other studies [26–28]. Therefore, no difference in variables such as BMI that needed long-term exercise to improve was found. Second, dietary pattern is critical to the success of management of weight. However, we did not monitor the dietary intake of participants because our aim was to compare the effectiveness of Tai Chi versus aerobic exercise. Third, we didn’t set blank control group in this study. However, previous studies have demonstrated that Tai Chi had beneficial effects on human body compared with control group. Moreover, our study demonstrated that Tai Chi was more effective in weight reduction, improving BP and HbA1c level in older persons with depressive symptoms compared to aerobic exercise. It suggested Tai Chi was indeed effective in improving BP and glucose control in older persons with depressive symptoms. Forth, there were many types of aerobic exercises, but we only compared Tai Chi to the low-impact aerobics, which was practiced widely. Further rigorous RCTs are warranted to compare the effects of Tai Chi with other types of aerobic exercise. Fifth, we did not follow up our participants to analyze the long-term effects of the 12-week Tai Chi intervention on weight, BMI, BP, and HbA1c level. A further study should be conducted to explore these long-term effects.

## Conclusions

The findings suggested that Tai Chi was an effective approach for management of BP and long-term glucose control.

## Data Availability

The datasets used and/or analyzed during the current study are available from the corresponding author on reasonable request.

## Abbreviations

BP: Blood pressure
HbA1c: Glycosylated hemoglobin
PHQ-9: Patient Health Questionnaire 9-Item
BMI: Body mass index
SBP: Systolic blood pressure
DBP: Diastolic blood pressure
